# A dataset of risky and ambiguous decisions using a novel Linked Colored Lottery Task across two studies

**DOI:** 10.1016/j.dib.2025.111844

**Published:** 2025-06-27

**Authors:** James B. Wyngaarden, Yi Yang, Jeffrey B. Dennison, David V. Smith

**Affiliations:** a Temple University, Philadelphia, PA, USA; b University of Pennsylvania, Philadelphia, PA, USA

**Keywords:** Probability, Variance, Ambiguity aversion, Belief updating

## Abstract

How individuals make decisions under ambiguity (i.e., uncertain situations where the probability of an outcome is unknown) has been related to numerous individual differences of clinical importance including aging, substance use, and autism spectrum disorders. Despite this, many studies rely on a max-min model of ambiguity decision-making, which assumes that individuals evaluate ambiguous options based solely on worst-case and best-case scenarios. However, this approach does not account for the role of individual beliefs about underlying probabilities, which can significantly shape decision-making behavior. We introduce a novel task, the Linked Colored Lottery Task, in an in-person (N= 53) and online sample (N= 300) which allows for analyses that examine the effect of these beliefs. Along with this novel task, demographic information and data for several personality and clinical questionnaires were collected. By incorporating measures that capture differences in probability beliefs, this dataset enables researchers to examine how ambiguity-related decision-making varies beyond max-min assumptions, providing a richer understanding of how individuals form and act upon beliefs about probabilities. These data offer opportunities to explore how individual tendencies—such as risk preferences, cognitive styles, and clinical traits–interact with beliefs about uncertainty, advancing both theoretical and applied perspectives on decision-making under ambiguity.

Specifications Table

**Table:** 

Subject	Social Sciences
Specific subject area	This area of research focuses on how individuals make decisions under conditions of risk and ambiguity
Type of data	experimental design information (tab-separated values [.tsv]) experiment meta-data (Java Script objection notation [.json])
Data collection	Experiment 1: Participants were brought into the lab after being consented and told they would be making a series of decisions about money performed on a laptop. The decision took the form of a two-alternative forced choice and programmed in PsychoPy. Participants were instructed that one of their decisions would be taken at random to determine a bonus payment. Experiment 2: Participants were recruited via an online recruitment platform, Prolific, and directed to an online experiment platform, Cognition.run where the experiment was hosted. Participants were then consented and presented with instructions for a two alternative forced choice decision task where they made decisions about money under conditions of risk, ambiguity, and limited feedback. Participants then completed a series of self-report questionnaires before being redirected back to Prolific.
Data source location	Institution: Temple University Location: Philadelphia, Pennsylvania, USA
Data accessibility	Repository name: Github Data identification number: https://doi.org/10.5281/zenodo.15122334 Direct URL to data: https://github.com/DVS-Lab/risk-ambiguity-DataInBrief

## 1 Value of the Data

• Provides opportunities for novel insights into how individuals integrate information about probability variance when making decisions under ambiguity, addressing a gap in existing research that has relied on max-min utility models.
• Offers a diverse sample with sociodemographic information (age, gender, ethnicity, education, zip code) and personality and clinical data, enabling robust analyses of individual differences in ambiguity preferences and their sociodemographic and psychological underpinnings.
• Includes reaction time and decision data suitable for computational modelling of decision-making processes, allowing researchers to test different model families beyond those considered by the original authors.
• Follows the BIDS (Brain Imaging Data Structure) format [1], facilitating easy navigation, integration, and potential combination with other datasets in this standardized structure.
• The inclusion of a feedback condition in Experiment 2 provides an opportunity to examine how direct experience with ambiguous lotteries influences subsequent decisions, offering a unique perspective on belief updating processes.

## 2 Background

Ambiguity aversion, the undervaluing of outcomes with unknown probabilities, has been linked to various psychological phenomena and clinical disorders [2,3]. Existing studies typically assume decisions are sensitive only to minimum and maximum probabilities, neglecting how individuals mentally represent ambiguous probabilities [4,5]. This overlooks a crucial aspect of decision-making under uncertainty: how people mentally represent ambiguous probabilities and incorporate them into their choices. Understanding these representations is essential for explaining individual variability in risk attitudes and linking ambiguity aversion to broader psychological and clinical factors. Without this additional information, links to psychological phenomena may be missed [6].

Previous tasks have included explicit manipulations of the distribution of probabilities, finding a general aversion to variance in probability distributions (e.g., [7]). However, these manipulations often include complex setups which poorly reflect real world conditions and may be difficult for some participants to interpret, potentially limiting ecological validity [8,9]. Our dataset introduces a novel implicit manipulation of probability distributions, enabling more precise exploration of individual differences in perceived ambiguity. By enhancing ambiguity aversion research methodologies, this work aims to clarify its cognitive mechanisms and significance in financial, medical, and social decision-making, building on a project examining neural responses to social and nonsocial rewards [10].

## 3 Data Description

We have organized the data records according to the BIDS (Brain Imaging Data Structure) standard (version 1.6.0), which is widely used within the fields of psychology and neuroscience [1]. Data from each study are organized in separate folders labelled Experiment 1 (in-person study) and Experiment 2 (online study). Each participant’s data are organized within participant-specific directories with the naming scheme <sub-XXX> and decision data are stored within a beh directory as *beh.tsv files. Participants’ reports from the memory questionnaire linked to the task will be labelled with the *beh.tsv and beh.json suffix.

Demographic information, including age, sex, ethnicity, education and zip code are detailed in the participants.tsv files (Exp 1 and Exp 2) and demographics.tsv file in a /phenotype directory placed at the root of the data directory (Exp 2 only). Other sets of participant level measurements are stored in the /phenotype directory placed at the root of the data directory for each study.

**Table:** 

Data Files	Description
beh_risk_amb_Exp1/sub-*/beh/sub-*_task-ambiguity_beh.tsv	Eight-column file describing the percent chance of winning the risky lottery, amount of money at stake, lottery color, level of ambiguity (i.e., percentage covered), whether the risky option is on the left side, response button, response time, and lottery distribution.
beh_risk_amb_Exp1/sub-*/beh/sub-*_task-risk_beh.tsv	Eight-column file describing the side of the lottery, percent chance of winning the lottery, amount of money for winning the lottery, lottery color, amount of money for choosing the non-lottery, lottery distribution, response button, and response time.
beh_risk_amb_Exp1/phenotype/*_win_count.tsv	Twelve-column file providing the reported win percentage for each probability (i.e., 0%-100%) across subjects from the memory questionnaire. One file for each lottery color (i.e., red, blue, green).
beh_risk_amb_Exp1/phenotype/yellow_seen_count.tsv	Twelve-column file providing the reported number of times yellow lotteries were associated with each probability across subjects from the memory questionnaire.
beh_risk_amb_Exp1/phenotype/accept_percentage_count.tsv	Twelve-column file providing the reported number of accept decisions for each probability across subjects from the memory questionnaire.
beh_risk_amb_Exp1/phenotype/ color_best_worst.tsv	Three-column file providing the reported best and worst lottery colors across subjects from the memory questionnaire.
beh_risk_amb_Exp2/sub-*/beh/sub-*_task-memory_beh.tsv	Five-column file describing the lottery color, lottery distribution, percent chance of winning the lottery, the number of times a participant reported seeing a given color lottery with the given chance of winning, and order of appearance for each lottery color.
beh_risk_amb_Exp2/sub-*/beh/sub-*_task-amb_run-*_beh.tsv	Ten-column file describing the block type, trial number, response time, lottery color, probability of winning the risky lottery, range of ambiguity, selected option, lottery distribution, side of the risky lottery, and response button.
beh_risk_amb_Exp2/sub-*/beh/sub-*_task-learn_run-*_beh.tsv	Eleven-column file describing the block type, trial number, response time, lottery color, probability of winning the risky lottery, risky lottery feedback, selected option, lottery distribution, side of the risky lottery, and response button.
beh_risk_amb_Exp2/sub-*/beh/sub-*_task-risk_run-*_beh.tsv	Nine-column file describing the block type, trial number, response time, lottery color, probability of winning the risky lottery, selected option, lottery distribution, side of the risky lottery, and response button.

## 4 Experimental Design, Materials and Methods

This dataset includes data collected from across two studies. Though the main questions of interest were the same across these studies, small changes to procedures have been made to optimize data collection. Where relevant we will detail the differences between studies.

### 4.1 Participants

Experiment 1: A sample of 46 participants [37 women, 9 men; age: mean (M) = 21.1 years, standard deviation (SD) = 1.8 years; 27 White, 14 Asian, 3 Black/African American, 2 Two or more races] completed Experiment 1 in person. Participants gave written informed consent as part of a protocol approved by the Institutional Review Board of Temple University. The same exclusion criteria were applied as described in the analysis plan of the main text [11]. Demographic information for participants in Experiment 1 have also been described in a previous publication (for full details see [11]; https://openneuro.org/datasets/ds004920/versions/1.1.1/metadata).

Experiment 2: For the second study data was collected online from a total of 287 participants [144 females, 142 males, 1 nonbinary; age: M = 45.2 years, SD = 15.5 years; 21 Asian or Pacific Islander, 34 Black or African American, 13 Hispanic, 1 Native American or Alaskan Native, 212 White or Caucasian, 6 multiple ethnicity]. Participants were pre-screened to through Prolific, an online crowdsourcing platform, so that the sample approximates recent data from the 2022 U.S. Census on demographics of age, sex, and ethnicities (see [12]). Participants gave written informed consent as part of a protocol approved by the Institutional Review Board of Temple University.

### 4.2 Procedures

Experiment 1: Data collected as part of Experiment 1 was collected from a convenience sample of individuals taking part of a larger neuroimaging experiment at Temple University Brain Imaging Center. All data presented here was collected during a “mock scan” session conducted one to two weeks prior to participants’ actual scan to assess suitability for neuroimaging. During this mock scan session participants were consented, performed the mock scan, completed the Linked Colored Lottery Task on a laptop provided to them, and answered a battery of questionnaires. Participants were informed that on the date of their scan they would be paid $25 per hour for their time inside the scanner and $15 an hour for their time outside of the scanner, plus a performance-based bonus of $0-$150 determined by their decisions across several tasks, including the Linked Colored Lottery Task described below.

Experiment 2: To facilitate a larger sample size, Experiment 2 data was collected online using the Prolific recruitment service. Prolific allowed us to compensate participants for their involvement ($6 for their participation) and link outcomes from one of their choices to a bonus they received after finishing the experiment (either $0, $3, or $7). The experimental protocol was hosted on the Cognition.run platform (https://cognition.run/). Participants completed informed consent, tasks, and questionnaires using their personal laptop or desktop computer in a place of their choosing.

#### 4.2.1 Linked Colored Lottery Tasks

We developed a novel set of linked decision tasks to investigate how ambiguous decisions may be influenced by individual beliefs about underlying probabilities. While the general form of the task is the same across both tasks, we detail any differences below. All task code has been made publicly available in our data repository, Experiment 1’s in-person task was implemented using the Python library PsychoPy [13], while Experiment 2’s online version used the JavaScript library jsPsych [14].

Our primary research question was whether participants could learn probability distributions in one context and apply that information when it was later hidden from them. To explore this, we developed two linked decision tasks: the Risky Colored Lottery task and Hidden Colored Lottery task. In the Risky task, participants learned about unique probability distributions associated with different colored lotteries while making decisions between known risks and safe amounts of money. In the subsequent Hidden task, participants made decisions about ambiguous lotteries where exact probabilities were concealed, but the lottery color remained visible—potentially allowing them to leverage distribution knowledge acquired during the Risky task. The following sections describe each of these tasks in greater detail.

#### 4.2.2 Risky Colored Lottery Tasks

This initial phase required participants to complete a series of two-alternate forced-choice decisions between a risky lottery and a safe amount of money. The risky lotteries offered a chance to win either a prize amount or $0. Lotteries were presented as pie charts with colored rings around them. The prize amount is displayed at the top and the probability of winning appeared at the bottom of each chart (see Fig. 2). Additionally, the probability of winning was visually represented by the bright region of the pie chart, while the dark region represented the probability of winning $0.

Throughout the task, the probability of winning varied while the prize money and safe amount remained constant. The task featured three lottery colors (red, blue, and green), with participants making 20 choices for each color before proceeding to the next. Critically, each color was assigned to one of three distinct probability distributions:

1. Wide distribution: Probabilities of winning clustered near the extremes (0% and 100%), decreasing in frequency as they approached 50% (see Fig. 1).

**Fig. 1:**
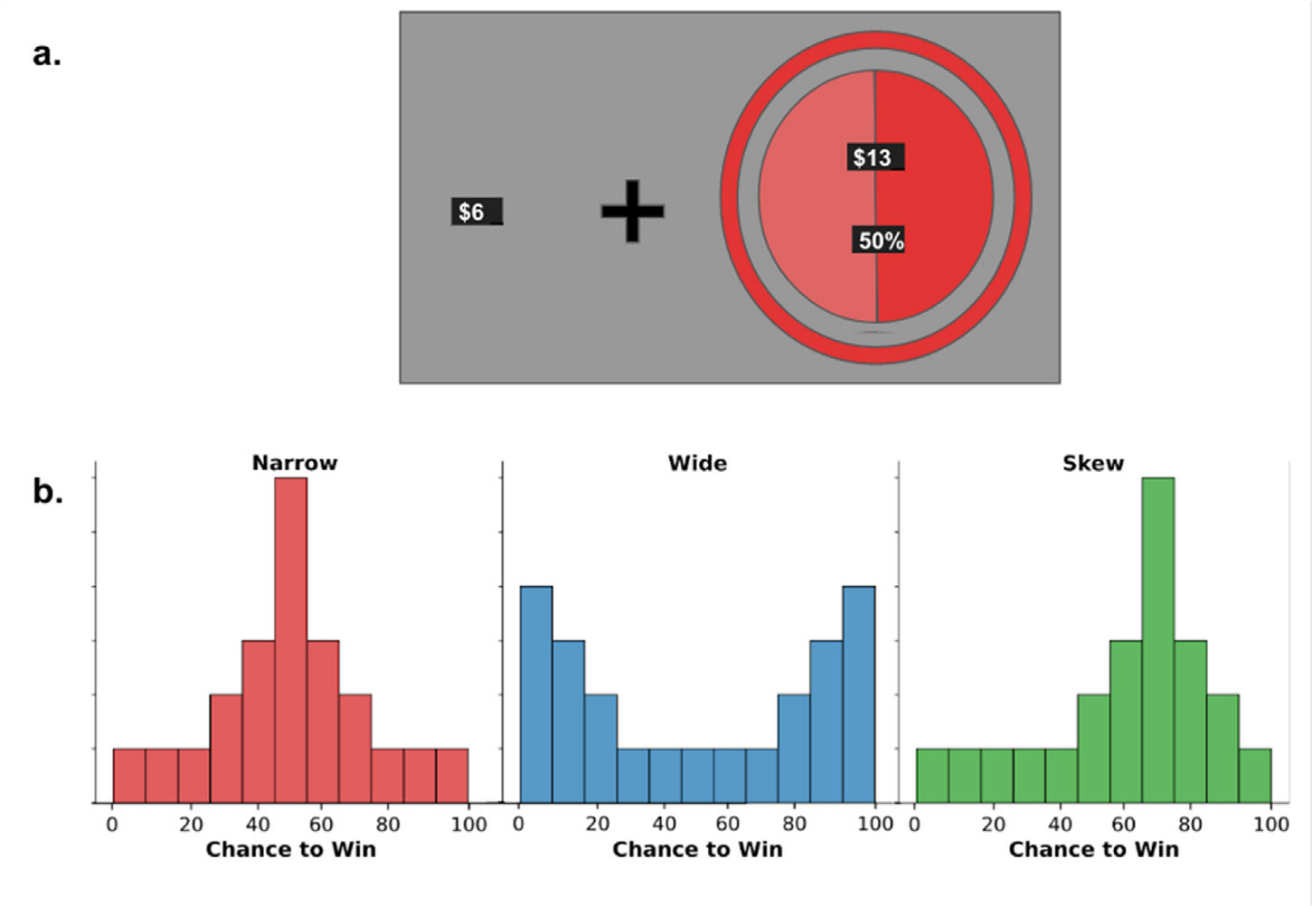
A schematic of the Risky Colored Lottery Task design. (a.) A visual representation of the two-alternative forced choice. Lotteries were randomly presented on either the left or right side of the screen. (b.) Three histograms representing how often color lotteries assigned a narrow, wide, and skewed distribution were presented with a particular chance of winning the prize of $13.
2. Narrow distribution: Probabilities most frequently centered around 50%, decreasing in frequency as they moved toward the extremes.
3. Skewed distribution: Probabilities most frequently centered around 70%, decreasing in frequency as they moved away from this point.

Minor procedural differences existed between experiments. In Experiment 1, the safe amount was $6 and the prize was $13, while Experiment 2 used $3 and $7, respectively. Additionally, Experiment 1 fully randomized the order of distributions, whereas Experiment 2 used one of four predetermined random orders (narrow-wide-skew, wide-narrow-skew, narrow-skew-wide, skew-narrow-wide).

#### 4.2.3 Hidden Colored Lottery Tasks

**Fig. 2:**
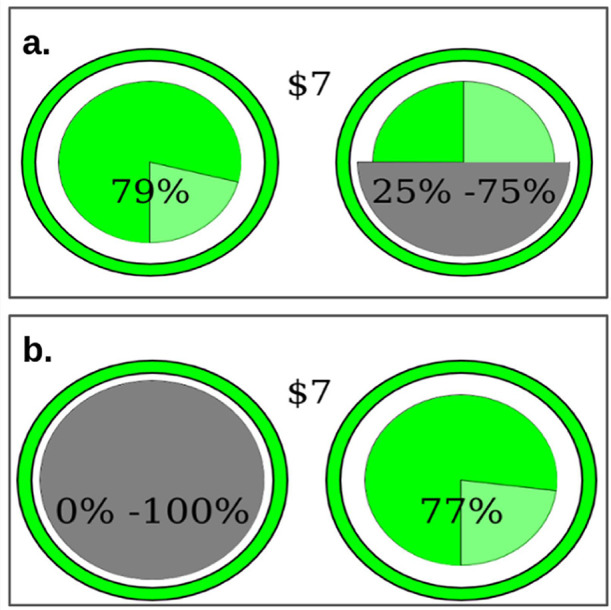
A schematic of the two-alternative forced choice for the Hidden Lottery Task. Each choice presents a risky lottery and hidden lottery randomly presented on the left or right. Like previous studies exploring ambiguity [15] the probability of winning is obscured either completely (a.) or partially (b.). However, the color of the ring around the lottery and portions of lottery that are un-obscured indicate whether the distribution the lottery is from (e.g., narrow, wide, or skewed).

In this second phase, participants again completed a series of two-alternative forced-choice decisions, but now chose between a risky lottery with known probability and a “hidden” lottery with ambiguous probability. The hidden lottery appeared as a pie chart with either full or partial occlusion, displaying a range of possible winning probabilities. Importantly, the color of the lottery ring remained visible, allowing participants to potentially infer the underlying probability distribution based on their experience in the Risky task (see Fig. 2).

Both experiments had procedural differences worth noting. In Experiment 1, participants made 8 choices for each color and occlusion amount, with the risky lottery probability ranging from 20% to 90% in 10% increments. In contrast, Experiment 2 featured 15 choices per color and probability range, employing an adaptive staircase procedure to determine the probability of winning the risky lottery. This procedure started with a 50% chance of winning, decreasing by 10% if participants chose the risky lottery and increasing by 10% if participants chose the ambiguous lottery. After two choice reversals, the step size halved (e.g., from 10% to 5%); after two identical choices, it increased by half (e.g., from 10% to 15%). These probability adjustments were bounded between 0% and 100%. Additionally, the prize was $13 in Experiment 1 and $7 in Experiment 2.

#### 4.2.4 Hidden Colored Lottery Tasks with feedback

Experiment 2 included an additional extension not present in Experiment 1: a version of the Hidden Lottery Task that incorporated feedback. In this variant, participants experienced a sampling period where they played the hidden lottery before making their decisions as described in the standard Hidden task. To play the lottery, participants pressed the space bar and received visual feedback: either a gold coin with “You won!” text or a grey coin with “You did not win” text. As in previous tasks, the lottery color (red, green, or blue) remained visible, allowing participants to incorporate this information alongside the feedback they received.

#### 4.2.5 Memory assessment

Experiment 1: Following the first lottery task, participants completed a memory assessment to evaluate their recognition and estimation of lottery characteristics. Participants were instructed to estimate the frequency of encountering lotteries of specific colors (e.g., red, green, blue) across varying chances of winning (0%-100%) by recording their best guesses in a provided table. They were also asked to identify which color lottery they perceived as the “best” and “worst” based on winning probabilities. Participants also rated, on a scale from 1 to 10, how frequently they believed they accepted lotteries with specific winning probabilities. Finally, participants were introduced to a novel lottery color (yellow) and asked to estimate how often they believed this lottery would represent each winning probability, using intuitive judgments as though it had been encountered during the task. Seven participants are missing memory assessment data.

Experiment 2: Participants completed a similar memory assessment. Participants estimated how often they recalled encountering each lottery (i.e., red, green, blue) paired with various probabilities of winning (e.g., 0%-100%) and categorized the probabilities accordingly. Additionally, participants indicated how frequently they associated each distribution with specific probabilities. The assessment also asked participants to recall the order in which they were exposed to each lottery, allowing for analyses of potential order effects on memory performance.

#### 4.2.6 Questionnaires

Experiment 1: Over the course of the experiment participants completed several questionnaires and inventories linked to reward processing, decision making, and clinical features of interest. This questionnaire data was made openly available as part of a previous publication (details about specific questionnaires are described in [11]; https://openneuro.org/datasets/ds004920/versions/1.1.1/metadata). Behavioral data from this experiment can be linked to these questionnaires via subject ID.

Experiment 2: After completing the Linked Colored Lottery Tasks, participants completed a comprehensive battery of questionnaires assessing demographic characteristics, autism spectrum traits, financial vulnerability, socioeconomic status, mood, substance use, and personal experiences with financial exploitation. Detailed descriptions of each measure are provided below. We note that all details about scoring are included in the corresponding .json files in the phenotype directories.

Demographic Information: Participants provided basic demographic information including age, gender, location (zip code), and education level.

Autism Spectrum Quotient (AQ): The AQ [16] is a 50-item self-report questionnaire designed to assess autism spectrum traits in adults, with 10 items for each of five theoretically derived subscales: social skill, attention switching, attention to detail, communication, and imagination. Participants respond using a 4-point Likert scale ranging from “definitely agree” to “definitely disagree”. Sample items include statements such as “I prefer to do things with others rather than on my own” and “I find social situations easy”. The AQ uses a binary scoring system, where an endorsement of the autistic trait (either mildly or strongly) is scored as a +1, while the opposite response is scored as a 0, leading to a maximum score of 50. Many items are reverse-coded during scoring so that higher scores consistently indicate greater autism spectrum traits. The measure demonstrated good internal consistency in the current sample (Cronbach’s α=0.82).

The Financial Exploitation and Vulnerability Scale (FEVS): The FEVS [17] is a 17-item self-report questionnaire designed to assess the risk of financial exploitation among older adults by evaluating contextual factors related to financial decision-making. The FEVS includes items that address domains such as financial strain, the impact of finances on social relationships, and worry about recent financial decisions. Participants respond to items using scales ranging from 0 to 2 or 0 to 3, depending on the item, with total scores ranging from 0 to 46; higher scores indicate greater vulnerability to financial exploitation. Risk levels are classified as low, moderate, or high based on the mean and standard deviation of FEVS scores within the sample. Sample items include statements such as “How often do your monthly expenses exceed your regular monthly income?”, “Has your relationship with a family member or friend become strained due to finances?”, and “How often do you worry about financial decisions you have recently made?”. The measure demonstrated good internal consistency in the current sample (Cronbach’s α=0.88).

The United States Index of Socioeconomic Deprivation for Individuals (USiDep): The USiDep [18] is an 8-item self-report questionnaire designed to assess individual-level socioeconomic deprivation in the United States. The USiDep includes yes/no questions addressing areas such as food affordability, heating costs, footwear condition, access to food assistance programs, and unemployment, with sample items like “Were you forced to buy cheaper food so you could pay for other essentials?” and “Have you often gone without fresh fruit and vegetables to save money?”. Responses are summed and converted to a 5-point ordinal scale (1–5), where higher scores indicate greater deprivation. The measure demonstrated acceptable internal consistency in the current sample (Cronbach’s α=0.73).

The 7 Up 7 Down Inventory: 7 Up 7 Down [19] is a 14-item self-report questionnaire developed to efficiently assess manic (7 Up) and depressive (7 Down) tendencies in adults and adolescents. The measure consists of two subscales, each containing seven items: the 7 Up subscale targets hypomanic and manic symptoms, while the 7 Down subscale assesses depressive symptoms. Respondents rate how often they experience each symptom using a 4-point Likert scale ranging from “never or hardly ever” to “very often or almost constantly.” Sample items include “Have you had periods of extreme happiness and intense energy lasting several days or more when you also felt much more anxious or tense than usual?” for the 7 Up subscale, and “Have there been times of several days or more when you were so sad that it was quite painful or you felt that you could not stand it?” for the 7 Down subscale. Scores are calculated by summing the responses for each subscale, with higher scores indicating greater severity of manic or depressive tendencies. The subscales both demonstrated acceptable internal consistency in the current sample (7 Up: Cronbach’s α=0.74, 7 Down: Cronbach’s α=0.76).

Substance Use Questionnaire: A questionnaire assessing recent drug use was administered based on a subset of items from the Adolescent Alcohol and Drug Involvement Scale (AADIS) [20]. This survey included 13 items evaluating the use of specific substances: tobacco products, alcohol, marijuana or hashish, hallucinogens, amphetamines, cocaine, barbiturates, PCP, heroin or other opiates, inhalants, and tranquilizers. Participants indicate their frequency of use for each substance on a 7-point scale ranging from 1 (never used) to 7 (several times daily), with higher total scores reflecting more extensive substance involvement. Sample items include “How often have you used alcohol (beer, wine, liquor)?” and “How often have you used marijuana or hashish (pot, weed, grass, blunts, wet)?”. As only a subset of AADIS items was administered, internal consistency reliability was not calculated for this version.

## Limitations

Key methodological differences between experiments limit direct comparability of results. Experiment 1 was conducted in a controlled laboratory environment during a mock scan session, while Experiment 2 utilized an uncontrolled online setting where participants used personal devices. This environmental variation may have introduced attentional differences and other confounds. Additionally, between-study differences in monetary incentives and specific task parameters like trial numbers could limit direct comparability across studies. Specifically, in-lab participants received larger incentives ($13 vs $7 prize; $6 vs $3 safe amount), which may have influenced risk preferences. The implementation of different probability determination methods (fixed increments vs. adaptive staircase) and distribution randomization approaches further complicates cross-experiment comparisons. The invariable task order—Risky task always preceding Hidden task—introduces potential order effects, while the feedback condition present only in Experiment 2 prevents certain cross-experiment analyses.

## Ethics Statement

All participants provided written informed consent to engage in the study and share their de-identified data publicly. The studies were approved by the Institutional Review Board at Temple University (Philadelphia, Pennsylvania, USA) under Protocol Number 28455 (Experiment 1) and Protocol Number 24452 (Experiment 2), and they were conducted in accordance with the Declaration of Helsinki.

## CRediT authorship contribution statement

**James B. Wyngaarden:** Writing – original draft, Writing – review & editing. **Yi Yang:** Data curation, Writing – review & editing. **Jeffrey B. Dennison:** Conceptualization, Methodology, Software, Formal analysis, Data curation, Writing – original draft, Writing – review & editing, Supervision, Project administration. **David V. Smith:** Conceptualization, Methodology, Writing – review & editing, Supervision, Project administration, Funding acquisition.

## Data Availability

ZenodoA Dataset of Risky and Ambiguous Decisions Using a Novel Linked Colored Lottery Task Across Two Studies (Original data).
